# Viewpoint: Next-Generation Sensor Technologies for Studying Mental Health

**DOI:** 10.1109/lsens.2026.3673618

**Published:** 2026-03-16

**Authors:** Christina H. Liu, Yvonne M. Bennett

**Affiliations:** National Institute of Mental Health, Division of Data Science and Technology, National Institutes of Health Rockville, Rockville, MD 20852 USA

**Keywords:** Chemical and biological sensors, aptamers, behavior, bioelectronics, biomarkers, brain, electrochemical sensing, mental health, neurotransmitters, nucleic acid sensing, organic transistors, sensors, wearable devices, wearables

## Abstract

Sensors of all kinds continue to be further refined and developed for a wide range of applications, including in the biomedical domain. Sensors are routinely used for vital sign tracking and motion. However, more work is needed to further enhance research efforts in the study of mental health. This letter highlights this area of bioelectronics and biosensing to encourage researchers to improve capabilities in electrochemical sensing—a gap area, to enable synchronous monitoring of brain activity and behavior, leading to new insights into brain circuit function and improving closed-loop therapies.

## I. INTRODUCTION

Sensors are ubiquitous in our environment with a multitude of commercial and consumer applications. These devices, when designed specifically for research purposes in the form of noninvasive or minimally invasive bioelectronic designs, can capture critical aspects of animal and human behavior. They can help us record and predict events such as movement initiation, relapse, cyclical behavior, for example, and thus enhance our understanding of basic behavior neuroscience and related areas. Sensors help us to track or predict events, transduce key biomarkers and even refine closed-loop therapies for mental health disorders. When combined and validated with brain recordings such as deep brain stimulation (DBS), electrocorticography, or responsive neurostimulation (RNS) systems for example, sensor data can also augment our knowledge of internal brain states. This article highlights programs at the National Institute of Mental Health (NIMH) that aim to develop state-of-the-art, next-generation sensors to enable us to record a range of signals about brain-behavior relationships and improve mental health. Specifically, development in electrochemical sensing is needed to help us identify and track neurochemicals involved in mental states.

## II. SENSORS PROGRAM AT THE NIMH

Several events including at the Institute of Electrical and Electronics Engineers (IEEE) Sensors Meeting in 2022 [1], and a presentation at the IEEE Body Sensor Networks in 2023 [2] highlighted the need for novel sensors and related biomarkers [3], [5], [6], to enable a transformative impact in our study of brain and behavior. A subsequent workshop at the National Institutes of Health (NIH), highlighted sensor technologies as part of the Brain – Behavior Quantification and Synchronization (BBQS) program [4]. The program is a component of the Brain Research Through Advancing Innovative Neurotechnologies (BRAIN) initiative that aims to further develop high-resolution tools, platforms, and theories to precisely quantify behavior as a multidimensional response, and to synchronize these data with simultaneously recorded brain activity. The research is focused on capturing naturalistic behavior, i.e., behavior that is untethered and freely-moving in a subject’s environment via wireless communication between devices or system components.

A funding opportunity entitled “BBQS - Next Generation Sensor Technology Development” has been published as part of this program and is open for applications (see RFA-MH-26–140, [7]). The Sensors Technology program invites researchers from a wide range of backgrounds to form multidisciplinary teams including: engineers, sensor developers, materials scientists, neurophysiologists, neurosurgeons, psychologists, psychiatrists, neuroethologists, computational neuroscientists, and data scientists to build next-generation sensor technologies and synchronize their data with brain recordings (either surface or intracranial) to establish models of brain – behavior relationships. The program encourages the development of novel sensors of all kinds, mainly in microelectromechanical systems and nanotechnology domains, and also invites quantum technology. Overall, systems-level frameworks for these large-scale projects with multiple sensors are highly dependent on the specific research application, but should strive to gather continuously streaming data of the highest spatial and temporal resolution possible on the order of days (48 h or more), for researchers to capture diurnal cycles of sensor data in real time. To further meet these goals, the following are also mandatory aspects in the design of next-generation sensors: high performance and accuracy of measurement beyond what has been previously demonstrated; low or zero power consumption via for example energy harvesting or high efficiency charging networks; high biocompatibility and low toxicity. Readers are encouraged to visit the Request for Applications (RFA) above for more information on this cooperative agreement.

## III. NEXT-GENERATION SENSOR TECHNOLOGIES: A FEW EXAMPLES

**Fig. 1.**
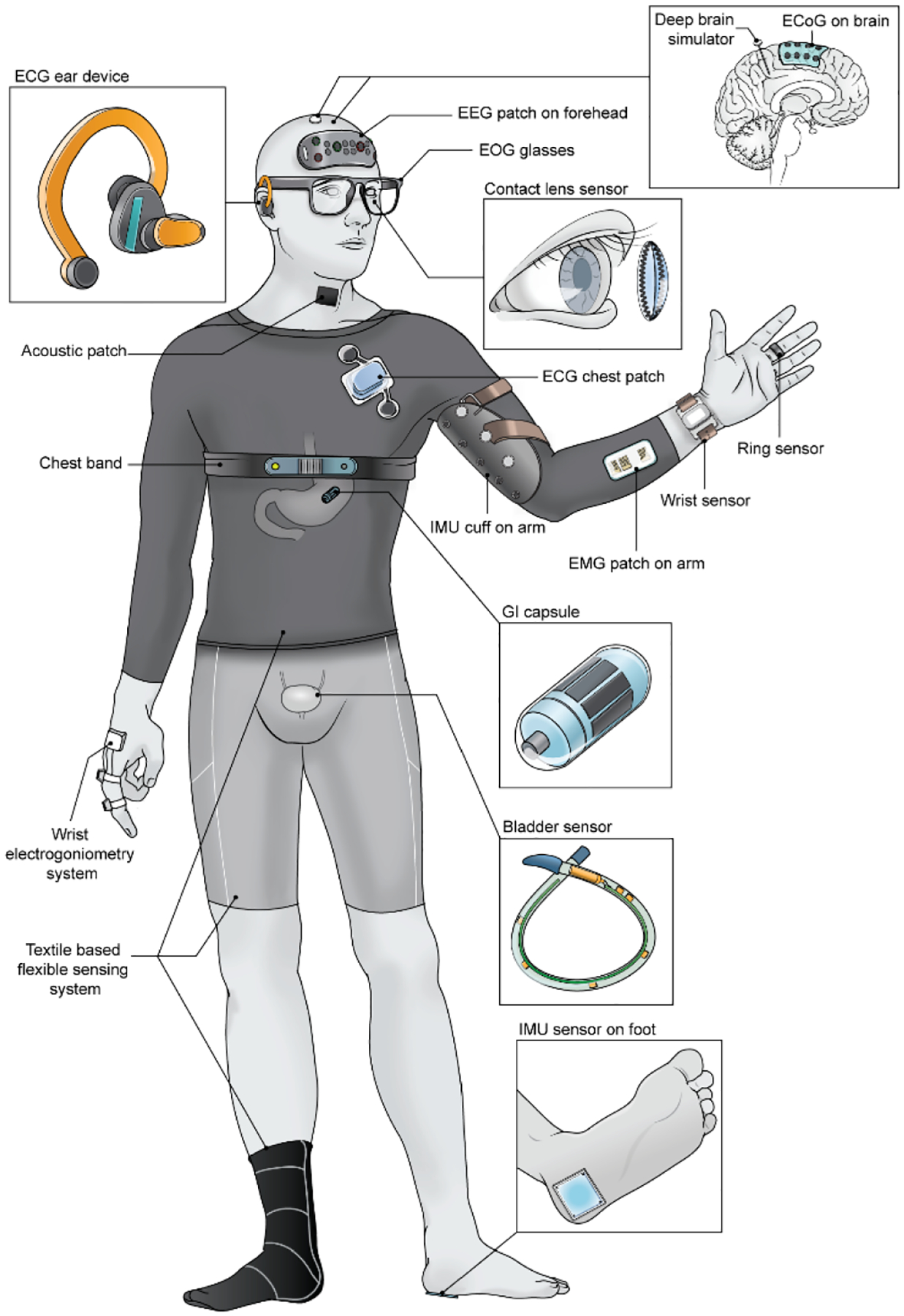
Examples of body-worn sensors, internal sensors, and brain recording devices, in a range of form factors with recent examples cited. A combination of multiple sensors will provide more complete information about the relationship between brain activities and behavior. In addition to data synchronization, challenges in multimodal integration and data analysis/computational modeling should be addressed. Sensors and brain recording systems: Deep brain stimulator (DBS) system [33], [35]; electrocorticography (ECoG) [40]; electroencephalography (EEG) patch on the forehead [41]; electrooculogram (EOG) system in glasses to track eye movements including saccades [42], [43]; contact lens sensor [44], [45]; earable multisensing device [46], [47]; textile-based garments with embedded thread sensors [9]; electrocardiogram (ECG) chest patch [47], [48]; inertial motion unit (IMU) cuff on the arm [49], [50]; electromyographic (EMG) patch on the arm [10], [50]; wrist and ring sensors [11], [50]; gastrointestinal internal (GI) capsule [52], [53], [54]; bladder sensor [55], [56]; IMU sensor on the foot for gait analysis [57], [58]; acoustic patch for speech/vocal recognition [23]; and electrogoniometry device to measure finger or wrist movements [11].

Current sensor types in the BBQS Sensors program include but are not limited to inertial, force/pressure, gaseous, optical, temperature, acoustic, remote, and electrochemical. Fig. 1 shows examples of various types of sensors deployed on or in the body. The examples in this section are mainly noninvasive or minimally invasive sensing, may be multimodal for example [8], and are by no means exhaustive in scope. A range of different form factors have been developed that can accommodate human or animal use, and may feature lightweight, flexible, or conforming designs, low or minimal power consumption, or have energy-harvesting capabilities, making them well-suited for continuous or long-term monitoring. Flexible electronics [9], [10], [11], [12] have the advantages of being comfortable while conforming to the body, the potential for capturing recordings for long periods of time, and incorporating multimodal sensing [8], [9], [10], [11].

*Inertial sensors* have been used to track and predict navigation and human gait, or can be used to monitor movement during the course of development in infants for example [13], [14].

*Force/Pressure sensors* were developed by Feng et al. [15] with the use of hydrogels for tactile sensing, and others using direct ink writing for MXene multifunctional tactile sensing [16]. MXene materials are a family of 2-D inorganic compounds which exhibit strong electrical conductivity, tunable layer spacing and versatile surface chemistry. A recent review of materials used for pressure biosensing is here [17].

*Gaseous sensors* [18] have recently been developed on a forearm design to measure transcutaneous oxygen noninvasively. Lim et al. [19] also reported a wearable luminescent oxygen sensor for transcutaneous oxygen monitoring using an organic photodiode as a light detector, there have been few effective methods for this type of sensing to date but spatial frequency domain imaging using near infrared technology holds promise as well [20].

A wearable sweat patch design for *optical sensing* of glucose, oxygen, and heart rate has been reported [21] via use of hydrogels and oxygen dyes that can be read as proportional to glucose concentrations, via wireless transmission and smartwatch monitoring.

A flexible *temperature sensor* has been developed for wearable system monitoring applications that can be used to track temperature changes in the body, using a skin-like conformable material composed of printed silicon nanoribbon [22]. This is a new system that tracks extreme temperature changes directly.

Wearable *acoustic sensors* offer a promising solution for effective communication for individuals with speech impairments by calibrating throat vibrations and digitizing speech. Wang et al. [23] developed a new type of piezoresistive acoustic sensor that is additively manufactured with polyurethane film-encapsulated graphene/cellulose nanocrystals via aerosol jet printing. The sensor is capable of measuring sound pressure levels from 30–90 dB with the use of support vector machine learning techniques.

A *remote* monitoring system for measuring breathing movements noninvasively via LiDAR with data privacy in place, has been developed [24]. This line-of-sight technology features a laser light system, with remote sensing and 3-D mapping measures that are not influenced by temperature, lighting, or object color and texture.

## IV. ELECTROCHEMICAL SENSING: FUTURE EMPHASIS IN MENTAL HEALTH DOMAIN

*Electrochemical sensing* [25] represents a relatively untapped area of research and development and is poised to make substantial contributions to our understanding of brain activity underlying mental health. Electrochemical sensing includes the detection of neurotransmitters such as dopamine, serotonin, and many other molecules, including metabolites, nutrients, electrolytes, and hormones. Neurotransmitters such as dopamine help us monitor mood states such as sadness, irritability, and anxiety and tracked with sleep patterns can help us detect these variables in clinical populations. Systems that were first developed in benchtop formats utilizing *aptamers* (short, single-stranded molecules of DNA or RNA that bind with high specificity to a target molecule) or *organic transistors* with single timepoints for detection in situ [28] track conformational changes in molecular entities. These systems are now moving to real-time and continuous recordings of molecules in blood, sweat, and interstitial fluid for example and are in wearable and ingestible forms [24], [25], [26], [27], [29].

*Ingestible capsules* for probing gut-brain interactions are being developed [26], [27]. Their features include sensing/sampling actuators, real-time data capture, and direct visualization of the entire gastrointestinal tract.

There are technical challenges in electrochemical sensing to be overcome with our ability to achieve continuous and real-time recordings, including the use of reagents versus nonreagent schemes, biofouling and related surface chemistry, stability of the redox probe over time, receptor design/binding affinity, and issues around the biofluidic sensor interface [25], [30]. Nevertheless, these electrochemical sensing designs hold great potential to record fluctuations of key molecules that will someday provide continuous measurements in real-time, and allow us to better track mental health status.

## V. CONCLUSION

The time is right to develop sensors and bioelectronics to augment the data streams in research aimed at improving our understanding of complex behavior and mental health. The NIMH is interested in supporting the development of sensors to track events and biomarkers during behaviors of interest, and in particular, the development of electrochemical sensors that will provide readouts of neurochemicals such as neurotransmitters, hormones, and metabolites related to internal brain states and their linkage to mental health. Our ability to continue to couple novel sensor data with brain recordings will bring forth new knowledge of how brain networks are involved in behavior through careful data analysis and development of computational models.

Information from computational models may provide insights into closed-loop therapies, which in the neurotechnology space are systems that use a feedback loop to monitor brain activity (such as electroencephalography (EEG) patterns) and automatically adjust neurostimulation to treat psychiatric and neurological disorders. This is in contrast to open-loop systems, which operate without feedback and deliver a predetermined set of stimuli regardless of outcome. Through the development and refinement of electrochemical sensor technologies and in combination with other sensor types, sensor data can aid in refinement of closed-loop therapies for mental health disorders. Closed-loop and adaptive therapies such as transcranial magnetic stimulation [31], [32], DBS [33], [34], and RNS systems may all stand to benefit and can improve mental health conditions [35], [36] including multiple brain regions [37]. The addition of sensor data streams will enable more precise localization of neural activity and personalized neuromodulation of corresponding neural circuits [38].

Applications involving the development of sensor technologies can be submitted via the BRAIN Initiative RFA-MH-26–140 [7] or via the NIH Parent announcements [39] in consultation with the authors.
